# C/Ni/N Nanocomposites Based on Hydrolysis Lignin: Synthesis, Study of Structural and Magnetic Properties

**DOI:** 10.3390/nano14231886

**Published:** 2024-11-23

**Authors:** Ihor Bordun, Dariusz Calus, Ewelina Szymczykiewicz, Myroslav Malovanyy, Nazar Nahurskyi, Anatoliy Borysiuk, Yuriy Kulyk

**Affiliations:** 1 Faculty of Electrical Engineering, Czestochowa University of Technology, J. Dabrowskiego Str. 69, 42-201 Czestochowa, Poland; dariusz.calus@pcz.pl (D.C.); ewelina.szymczykiewicz@pcz.pl (E.S.); 2 Viacheslav Chornovil Institute of Sustainable Development, Lviv Polytechnic National University, Bandera Str. 12, 79013 Lviv, Ukraine; myroslav.s.malovanyy@lpnu.ua (M.M.); nazar.o.nahurskyi@lpnu.ua (N.N.); anatoliy.k.borysyuk@lpnu.ua (A.B.); 3 Faculty of Physics, Ivan Franko Lviv National University, Universytetska Str. 1, 79005 Lviv, Ukraine; yuriy.kulyk@lnu.edu.ua

**Keywords:** carbon nanocomposites, porous structures, superparamagnetic particles, residual magnetization, phase magnetic analysis

## Abstract

A two-step method for the synthesis of C/Ni/N nanocomposites based on hydrolysis lignin from wood chemical processing waste is proposed. These nanocomposites were found to have a well-developed porous structure with a wide pore size distribution. It was shown that doping hydrolysis lignin with urea-derived nitrogen leads to the appearance of ferromagnetic behavior in the carbon material. When nickel chloride was added during pyrolysis, the magnetic behavior of the C/Ni/N composite was provided by superparamagnetic Ni particles less than 30 nm in size and the magnetism of the carbon matrix. The addition of urea during the synthesis of the nanocomposite further promotes better integration of nickel into the carbon structure. According to the results of magnetic studies, the nickel content in the C/Ni/N nanocomposite was 19 wt.% compared to 15 wt.% in the C/Ni nanocomposite. The synthesized nanocomposite was demonstrated to have no residual magnetization, so its particles do not agglomerate after the external magnetic field is removed. Due to this property and the well-developed porous structure, C/Ni/N composites have the potential to be used as catalysts, active electrode materials for autonomous energy sources, and in environmental technologies as magnetically sensitive adsorbents.

## 1. Introduction

The unique properties of carbon materials (hydrophobicity, large specific surface area and volume, hydrolytic and thermal inertness, etc.) combined with their ease of production and low production costs make them widely used in various fields of human activity. These include adsorbents, carriers of catalytically active substances, active materials for power supply electrodes, ion exchange materials and many more [1,2,3,4,5,6,7,8]. However, standard synthesis methods using pyrolysis and various activators do not allow to obtain materials that would combine both versatility and high efficiency for certain processes. Therefore, in recent years there has been an increasing development of synthesis methods for porous carbon materials with specific functionalization through chemical and structural modification. One of the key methods is the synthesis of such materials by incorporating other chemical elements into the carbon matrix. This synthesis results in either a metal-carbon composite or a carbon structure with additional functional groups on its surface [9,10,11,12,13].

Among such materials, nickel/carbon composites deserve great attention, as they can be used in various processes. These composites have demonstrated high efficiency, a broad absorption band, and environmental safety in the remediation of electromagnetic radiation pollution [14,15]. Environmental protection should also be accompanied by the transition to fossil fuel alternatives. Hydrogen is one such fuel. Hydrogen can be produced in several ways, from classical electrolysis to dehydrogenation of a solid. Nickel and carbon-based composites are important for such technologies. Nickel and nickel-based composites act as co-catalysts in photocatalytic hydrogen production reactions. These composites have structural stability, a simple synthesis methodology, and high performance in the hydrogen production reaction [16,17]. Hydrogen can also be produced from solids such as hydrides, alanates and/or borohydrides, e.g., lithium or magnesium. They hold more hydrogen with a higher density than compressed or liquid hydrogen. Thermodynamic and kinetic limitations to the efficient use of these solid materials can be overcome by the addition of C/Ni composites [18,19,20]. Another potential application of C/Ni composites is as active materials for supercapacitor electrodes. Such composites exhibit high specific capacitance, good electrochemical parameters and the ability to withstand a large number of charge/discharge cycles [13,21,22]. In water purification systems, carbon materials are the main type of adsorbents for various pollutants. However, treating large volumes of water always involves removing fine adsorbent particles from the treated medium. One of the ways to solve this problem is to use magnetic separation. To achieve this, the carbon adsorbent is modified with an activator that makes it sensitive to magnetic fields. C/Ni composites are among such magnetically sensitive adsorbents [23,24].

Another chemical element that changes the properties of the carbon matrix in the desired direction is nitrogen. The addition of nitrogen allows such a carbon material to be used as an effective material for the manufacture of electrodes for power sources and electrical desalination systems [12,25,26,27]. Nitrogen doping improves the adsorption properties of the carbon material both for different types of pollutants [28,29] and for the adsorption of carbon dioxide [30].

The combination of these two chemical carbon matrix modifiers would make it possible to obtain a carbon composite with even more promising properties. Such composites are also effective for the fabrication of supercapacitor electrodes [31], absorption of microwave radiation [32], or as highly efficient electrocatalysts for the methanol oxidation reaction in fuel cells [33]. However, the synthesis of such composites is not straightforward and requires the use of either a properly formed metal-organic framework [31,32] or nickel-coordinated polyaniline-polyvinyl alcohol hydrogels [33].

Our previous studies have shown the fundamental possibility of synthesizing nanostructured C/Ni composites based on bio-carbon materials by pyrolysis with steam-gas activation [34,35]. Therefore, this study aimed to develop a methodology for the synthesis of C/Ni/N composites from natural raw material processing wastes to investigate their structure, porosity, and magnetic properties.

## 2. Materials and Methods

### 2.1. Selection of Raw Materials and Methods for the Synthesis of Nanocomposites

The active development of various industries has led to the generation of significant amounts of solid waste that seriously pollutes the environment. Therefore, there is an important problem of recycling such waste or obtaining useful products from it. Hydrolysis lignin is one of the most common industrial wastes from wood chemical processing plants. Many technologies have been developed to produce renewable fuels, chemical compounds, or adsorbents from it [36,37], but the utilization of such waste is still insufficient. Carbon composites or fibers synthesized from lignin are widely used [38,39,40], so this area of research is promising for the development of new carbon materials. Based on these considerations, hydrolysis lignin was chosen as a raw material for the synthesis of C/Ni/N nanocomposites.

**Figure 1:**
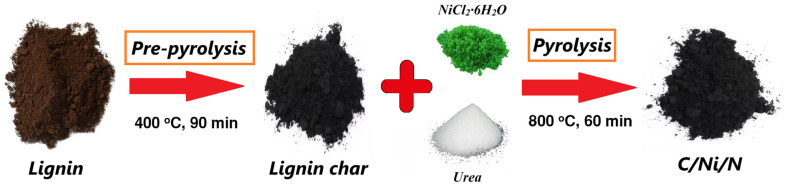
General scheme for the synthesis of nanocomposites C/Ni/N.

Studies [34,35] have shown that a two-step synthesis is effective in developing the porous structure of the resulting carbon material. A general scheme for the two-step synthesis of nanocomposites is shown in Figure 1.

In the first step, lignin was pre-pyrolyzed. The lignin was placed in an oven and the pre-pyrolysis was carried out at a temperature of 400 °C for 90 min. In the second step, the obtained char was mixed with NiCl_2_·6H_2_O and urea (for nitrogen doping) in a mass ratio of 0.75:1:0.5, the required amount of distilled water was added and the mixture was stirred under heating until the NiCl_2_·6H_2_O and urea were completely dissolved. The mixture was dried at 110 °C. The dried powder was placed in a ceramic crucible and heated in a furnace at a rate of 10 °C/min to a temperature of 800 °C in an argon stream and held at this temperature for 60 min. The resulting nanocomposite was washed and dried at 110 °C in an oven. These samples are hereafter referred to as C/Ni/N composite.

To ascertain the impact of each precursor on the characteristics of the composite, a C/Ni nanocomposite devoid of nitrogen and a C/N carbon material doped with nitrogen were also synthesized. The synthesis was also conducted in two stages and under the same conditions. The corresponding ratios between the precursors used in the synthesis of the samples are given in Table 1.

### 2.2. Experimental Research Methods

The morphology of the nanocomposites was investigated using scanning electron microscopy (SEM) using a Phenom ProX microscope (ThermoFisher Scientific, Waltham, Massachusetts, USA) with an energy dispersive X-ray (EDX) system for determining the elemental composition of particles. EDX microanalysis was conducted at points on the surface of the nanocomposite particles at an operating voltage of 15 kV.

X-ray diffractograms of the carbonaceous materials were obtained using a DRON-3 diffractometer with a wavelength of λ = 0.1542 nm (Cu K_α_-radiation, which was monochromatized by reflection from the plane (002) of a single pyrographite crystal). Diffractograms for all samples were obtained in continuous scanning mode at an angular velocity of 2° per minute over a diffraction angle range of 2*θ* = 5° to 105°.

Given the complex and heterogeneous (porous) structure of the synthesized composites, the method of small-angle X-ray scattering (SAXS) was employed as an additional X-ray investigative technique. SAXS spectra were obtained using a DRON-3 diffractometer with monochromatic Cu K_α_-radiation. The samples were placed in powder form within 1.5 mm high cuvettes. The cuvettes’ windows were covered with a 50 µm thick polyethylene film. Before analysis, a correction for background scattering and a collimation correction for the height of the detector’s receiving slit were applied to the scattering intensity curves.

To study the parameters of the porous structure, the method of adsorption/desorption of nitrogen at its boiling point was employed. The measurements were conducted using an automated analyzer, the Quantachrome NOVAtouch LX2 (Anton Paar QuantaTec, Boynton Beach, FL, USA). Before measuring the adsorption/desorption isotherms, all carbon materials were degassed in a vacuum at 473 K for 16 h.

Magnetic measurements were performed using a vibrating sample magnetometer (VSM) of our design, described in detail in [41]. Magnetic moment hysteresis loops were recorded in a magnetic field with a strength of −300 kA/m to +300 kA/m. The measurements were conducted at room temperature. Specific saturation magnetization and its temperature dependence were measured at a magnetic field strength of 800 kA/m. Before the measurements, the magnetometer was calibrated using a comparison process, employing a reference sample of pure, non-porous nickel with a density of ρ = 8.9 g/cm^3^ and a specific magnetization of 54.8 A·m^2^/kg.

## 3. Results and Discussion

### 3.1. Particle Morphology and X-ray Diffraction of the Synthesized Carbon Materials

**Figure 2:**
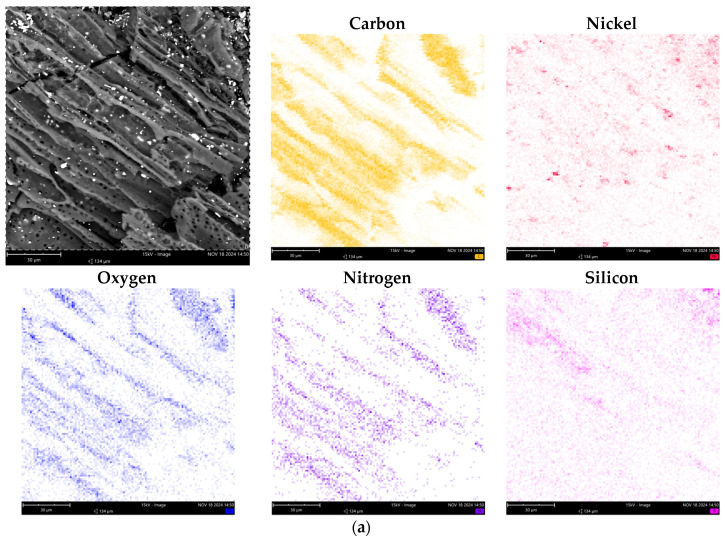

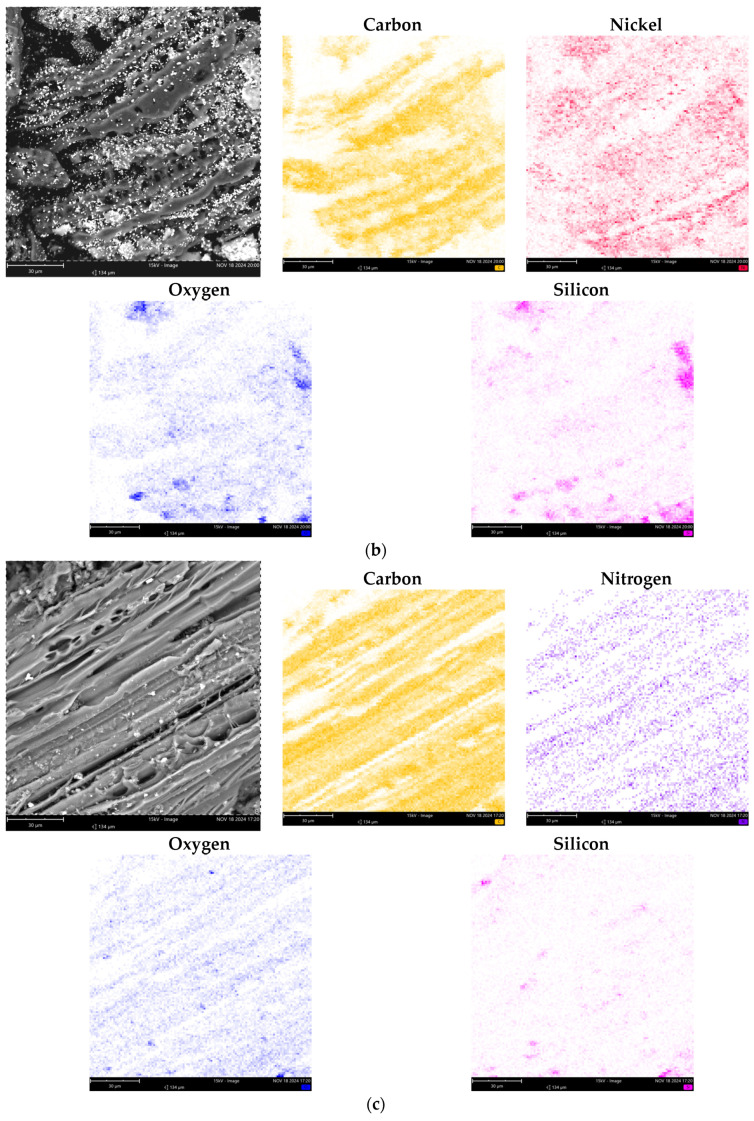
SEM images and EDX-mapping of the chemical element distribution of C/Ni/N (**a**), C/Ni (**b**) and C/N (**c**) samples.

The SEM images of all synthesized composites are similar (Figure 2a–c). The composites are primarily composed of carbon particles that retain the structure of the original hydrolysis lignin particles derived from plant raw materials.

To further characterize the synthesized nanocomposites, EDX mapping of the particle surfaces was performed, as shown in Figure 2. The average content of chemical elements is provided in Table 2. As can be seen, the composition is predominantly composed of carbon atoms. Additionally, nickel and nitrogen atoms are fairly uniformly distributed throughout the structure of the synthesized composites (Figure 2). The concentration of nickel atoms in the C/Ni/N composite is slightly higher than in the C/Ni composite.

**Figure 3:**
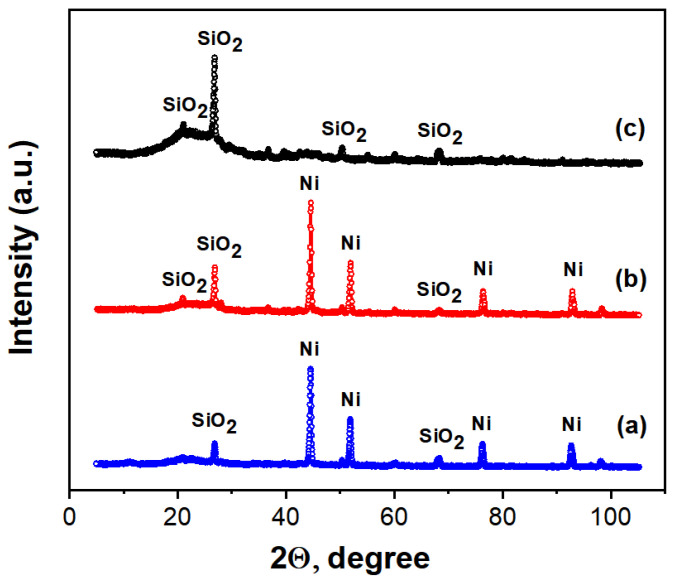
X-ray diffraction patterns of the synthesized nanocomposites C/Ni/N (**a**), C/Ni (**b**), and C/N (**c**).

The X-ray diffractograms of the synthesized samples are shown in Figure 3. The determination of the profile and structural parameters of the phase components in the diffractograms was carried out using the Jana2006 full-field analysis software package. The Lorentz function described the profile of the diffraction maxima.

A three-phase amorphous-crystalline structure is visible in the X-ray diffraction pattern of the C/Ni/N nanocomposite shown in Figure 3a. In addition to the amorphous carbon phase, Nickel and Silicon oxide phases can be identified in the sample.

Similarly, three phases were identified in the C/Ni nanocomposite, while two identified phases characterize the C/N sample. The characteristics of the identified phases are presented in Table 3. It should be noted that the amorphous carbon phase of all synthesized nanocomposites has a maximum located at an angle of 2θ ≈ 24.0°. The position of this maximum corresponds to the interlayer distance between graphene layers of d ≈ 0.37 nm. This indicates a certain carbon structure disorder since the distance between graphene layers is d ≈ 0.335 nm for crystalline graphite.

As can be seen from the comparison of these X-ray diffraction patterns, there is a difference in the additional carbon components, while the amorphous carbon component remains almost identical for all carbon materials. It is known [42] that graphite can reduce nickel oxide at temperatures above 900 °C. However, as can be seen from the XRD patterns in Figure 3, the synthesized carbon materials exhibit an amorphous carbon phase. Since amorphous carbon is more reactive, nickel oxide reduction occurs at lower temperatures, around 450–500 °C [43,44]. The silica impurity likely leached into the hydrolysis lignin during wood processing at the hydrolysis plant.

### 3.2. Porous Structure of Synthesized Carbon Materials

Considering the complex structure of synthesized nanocomposites, SAXS provides additional structural information. This X-ray scattering is caused by the formation of electron density distribution inhomogeneities in the material. Therefore, small-angle (X-ray or neutron) scattering is one of the most effective methods to study the atomic structure of a substance in the size range of 1 to 100 nm [45,46].

**Figure 4:**
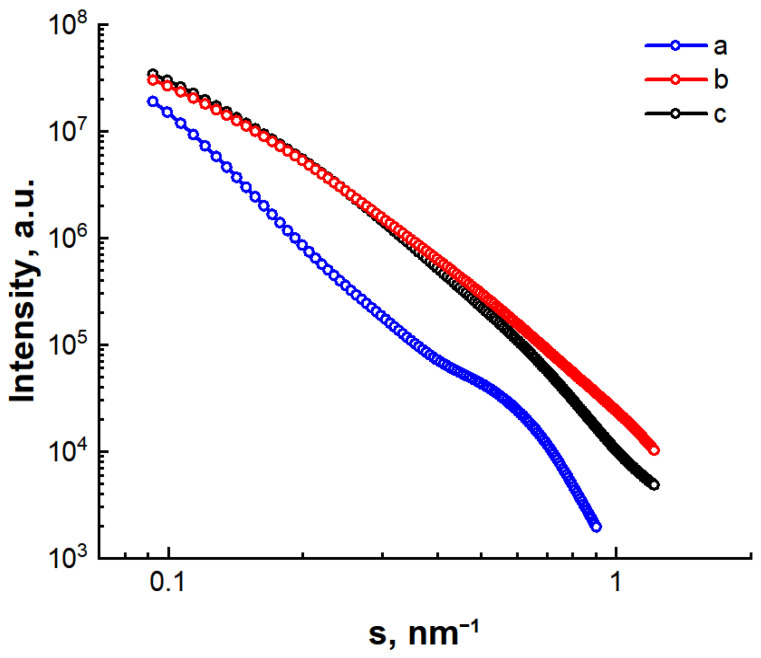
SAXS spectra of synthesized nanocomposites C/Ni/N (**a**), C/Ni (**b**) and C/N (**c**) (points—experimental data, solid line—smoothed curve).

The SAXS spectra of the synthesized C/Ni/N, C/Ni and C/N samples are shown in Figure 4 in double logarithmic coordinates after correction for absorption and detector slit height. Due to their size, the crystalline phases detected by XRD analysis are expected to have little effect on the scattering in the small angle range (0.1–2.0°). As can be seen from Figure 4, there is a monotonic decrease in scattering intensity over the entire angular range. This character of the curve indicates a random distribution of scattering inhomogeneities or pores.

In the SAXS spectrum of the C/Ni/N nanocomposite, three regions can be distinguished with different patterns of scattering intensity changes depending on the modulus of the wave vector *s* (Figure 4a). In the interval 0.15 < *s* < 0.35 nm^−1^, we observe a linear plot with a slope of *n* = 3.9, indicating a power law dependence of the intensity on the *s* module:

(1) $$I(s)\sim s^{-n}$$

The value of the index is close to n = 4, which corresponds to Porod’s law [47] for scattering by surface inhomogeneities with an almost smooth interface between the pores and the carbon base of the material. The typical sizes of the inhomogeneities (*L* ≈ 2 *π*/*s*), scattered by them in this region, vary in the range 18 < *L* < 42 nm. At the same time, a significant deviation from the linear decay of the scattering intensity is observed in the interval *s* > 0.35 nm^−1^. Such a change in intensity can be described by the Guinier formula, which corresponds to scattering by monodisperse inhomogeneities with a radius of inertia *r_g_* [48]:

(2) $$I\left(s\right)=I\left(0\right)exp\left(-\frac{1}{3}s^{2}r_{g}^{2}\right)$$

According to the calculations, *r_g_* = 4.1 nm, indicating the mesoporous structure of the C/Ni/N nanocomposite. Finally, a deviation from the linear dependence of the intensity on the scattering vector modulus is observed in the initial part of the curve *s* < 0.15 nm^−1^. It can be assumed that in this interval scattering is observed from large inhomogeneities with a radius of inertia *R_g_* ≈ 25 nm, which is also described by the Guinier Formula (2).

**Figure 5:**
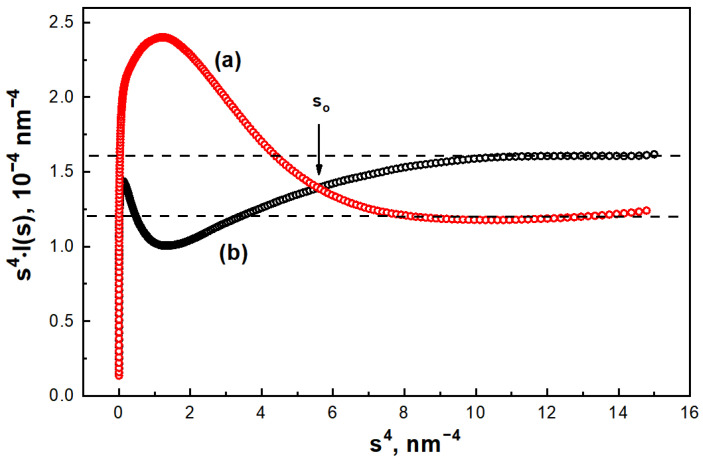
SAXS curves plotted in Porod coordinates *s*^4^*·I(s) = f(s*^4^*)* for C/Ni (**a**) and C/N (**b**) samples.

The SAXS spectra for the C/Ni and C/N carbon materials are non-linear, practically coinciding up to wave vector values s < 0.3 nm^−1^, and then there is a slight difference. It is difficult to distinguish any regions in these plots, so for a deeper analysis we additionally measured the SAXS intensity curves for C/Ni and C/N samples for the range of wave vector values *s* = 1 ÷ 2 nm^−1^. The spectra obtained were reconstructed in Porod’s coordinates *s*^4^*·I(s) = f(s*^4^*)*. As can be seen from Figure 5, for both samples the behavior of the intensity curves at s→∞ is satisfactorily approximated by Porod’s law (*I(s) ~ s*^4^), which, as in the case of the C/Ni/N nanocomposite, indicates the formation of a smooth interface between the pores and the carbon matrix. Notably, the *s*^4^*·I(s*) dependencies of the C/Ni and C/N samples behave differently for the wave vector intervals values *s < s_o_* and *s > s_o_*, where *s_o_* ≈ 1.5 nm^−1^ is the point of intersection of the Porod curves (Figure 5).

Thus, for the C/N sample, an increase in scattering intensity is observed in the *s > s_o_*, angular interval, indicating a dominant contribution to scattering by microscopic pores whose characteristic size does not exceed $L\approx \frac{2\pi }{s_{0}}\approx 4.0$ nm.

Instead, the opposite pattern is observed in the C/Ni sample: a significant increase in intensity is observed in the small scattering angle region *s > s_o_*. Thus, the small angle scattering of the C/Ni sample is mainly due to the formation of mesoscopic pores with an effective size of *L* > 4.0 nm. Since both samples have a smooth pore surface, their porous structure can be considered, to a first approximation, as a polydisperse system of spherical pores.

**Figure 6:**
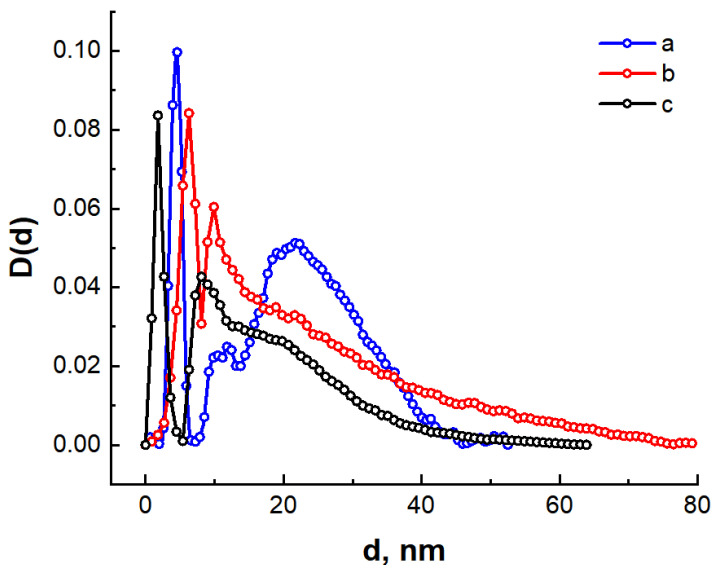
Volume distribution functions of effective pore diameters for C/Ni/N (**a**), C/Ni (**b**) and C/N (**c**) samples.

Figure 6 shows the volume distribution functions of pores in the synthesized carbon materials obtained by the indirect Fourier transform method for the spherical pore model. The results of the pore size distribution calculation correlate with the analysis of the scattering intensity curves.

For the C/Ni/N nanocomposite, a narrow maximum is observed on the curve corresponding to scattering by mesoscopic pores with an effective size of d ≈ 4.5 nm (Figure 6a). In addition, two maxima at d ≈ 11 nm and d ≈ 21.5 nm can be distinguished on this curve, indicating the formation of mesopores with a wide distribution of effective diameters in the range from 7.5 to 45 nm. The formation of a microporous structure is not observed in the C/Ni sample. Two distinct maxima are observed on the pore diameter distribution function, indicating the main contribution to scattering by mesopores with effective diameters of 6.3 and 10.0 nm. It should be noted that the C/Ni sample also contains larger mesopores with a wide diameter distribution from 15.0 to 75.0 nm. The volume distribution of inhomogeneities in the C/N sample also shows two maxima corresponding to effective pore diameters of 1.8 nm and 8.0 nm. The presence of an isolated maximum at 1.8 nm indicates the formation of microscopic pores. In addition, the formation of mesopores with a wide distribution of effective diameters from 15.0 to 60.0 nm is also observed in the C/N sample.

**Figure 7:**
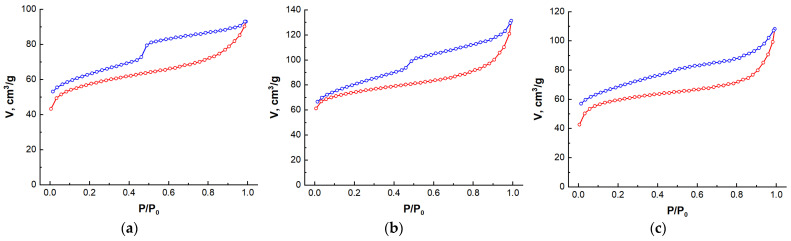
Nitrogen adsorption/desorption isotherms for the C/Ni/N (**a**), C/Ni (**b**) and C/N (**c**) samples.

Consequently, the small-angle scattering method enables the identification of the distinctive characteristics of the porous structure of the carbon materials under investigation. Nevertheless, this method enables the identification of not only open pores but also those that are closed. Such pores will not be involved in, for example, adsorption or catalysis processes. Moreover, if the structure of a carbon material contains a certain number of nanoscale objects with different electron densities, they will also scatter X-rays. To determine the available porous structure accessible to external ions and molecules, nitrogen adsorption/desorption processes at a temperature of T = 77 K were studied. The isotherms obtained for the synthesized nanocomposites are shown in Figure 7.

Different types of adsorption isotherms are observed depending on the structural characteristics of the material under investigation, of which Brunauer identified five main types [49]. The isotherms observed for the C/Ni/N, C/Ni, and C/N samples approximate type II, as illustrated in Figure 7. Furthermore, all isotherms exhibit distinctive hysteresis in the region of high relative pressures and a discrepancy between the adsorption and desorption curves in the region of low relative pressures. This isotherm behavior is indicative of capillary condensation within the mesopores.

To calculate the parameters of the carbon materials, the obtained isotherms were analyzed using Quantachrome TouchWin version 1.12. In particular, the Brunauer, Emmett, Teller (BET) multipoint method [50] was used to determine the specific surface area, which approximates the experimental data with a straight line in the range of relative pressures P/P_0_ = 0.05 ÷ 0.3. The BET theory is based on the kinetic model of the adsorption process proposed by Langmuir. In this model, the surface of a solid is considered as a set of adsorption sites. In a state of dynamic equilibrium, the rate of condensation of gas phase molecules onto free centers is equal to the rate of evaporation of molecules from the occupied sites. Although such a model has been repeatedly criticized, the equation proposed by these authors (the BET equation) makes it possible to determine the specific surface area, especially when the adsorption isotherm is of type II.

The calculations of the porous structure parameters are presented in Table 4. As can be observed, both activators do not result in any notable alterations or developments in porosity, while the use of Nickel Chloride alone leads to an increase in both the specific surface area and the specific volume in the C/Ni sample, compared to the other samples.

**Figure 8:**
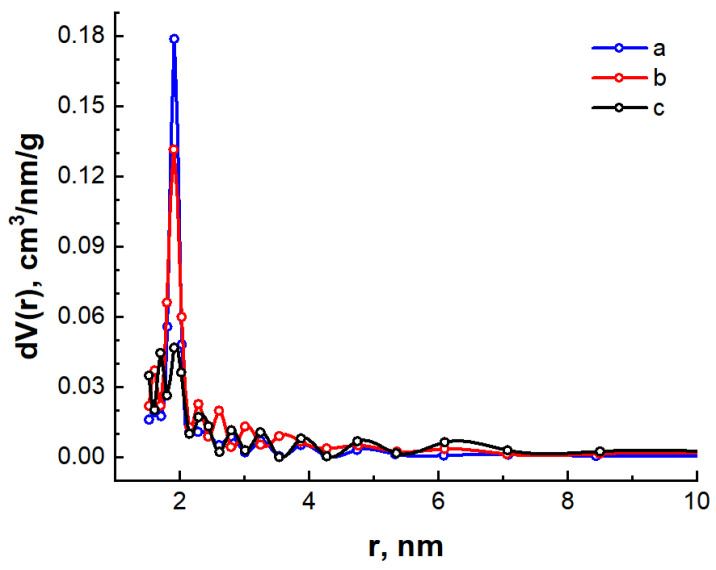
Pore size distribution for C/Ni/N (**a**), C/Ni (**b**) and C/N (**c**) samples calculated by the BJH method.

From the analysis of the SAXS spectra, it was concluded that the synthesized carbon materials possess a high content of mesopores in the porous structure, and the C/N sample also has a significant portion of micropores. For mesoporous materials, the BJH (Barret-Joyner-Halenda) method, which is based on the Kelvin equation, is often used to determine the pore size distribution [50]. To calculate the pore distribution using the BJH method, we used desorption isotherms that are closer to the true thermodynamic equilibrium. The calculation results are shown in Figure 8 and Table 4.

The graphs shown in Figure 8 are slightly different from those shown in Figure 6. For the C/Ni/N and C/Ni samples, there is a large peak in the region around the pore radius of 2 nm. For the C/N sample, the mesopores exist in a fairly wide size range, resulting in a larger total pore volume with a smaller specific surface area (Table 4).

**Figure 9:**
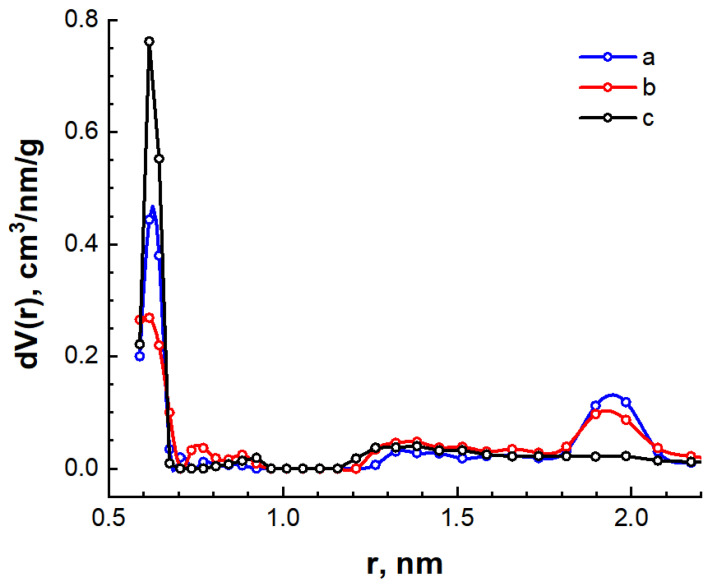
Distribution of pores by size for C/Ni/N (**a**), C/Ni (**b**), and C/N (**c**) samples calculated by the MP method.

The micropore structure was estimated using the MP method, which constitutes a modification of the classical *t*-method [50]. The results of the micropore parameter calculations are presented in Table 4, and the distribution of micropores by size is illustrated in Figure 9.

The micropore analysis yielded results that were corroborated by the small-angle X-ray scattering study. As anticipated, the C/N carbon material synthesized using only urea exhibits the highest micropore content. Therefore, the incorporation of urea results in an enhancement of the micropore content of the C/Ni/N sample relative to the C/Ni sample. The total specific area and volume of micropores and mesopores was found to be slightly lower in comparison to the BET data. This discrepancy may be attributed to the underestimation of these parameters for mesopores in the BJH calculation, given that the capillary condensation of nitrogen occurs in mesopores, which influences the isotherm shape of the isotherm.

Therefore, the assessment of the porous structure of the synthesized carbon materials revealed that all samples exhibited porosity in the micro- and mesopore regions.

### 3.3. Magnetic Properties of Synthesized Carbon Materials

All synthesized composites, including those free of metal atoms, exhibit ferromagnetic behavior at room temperature.

**Figure 10:**
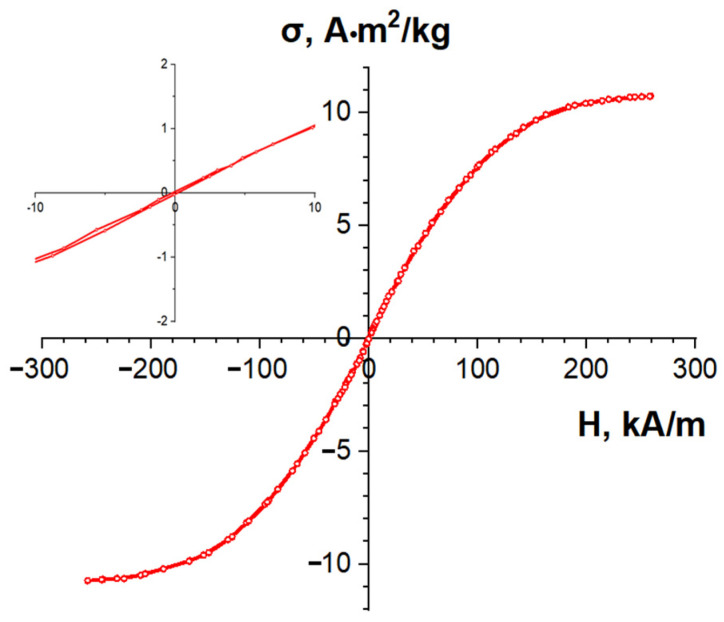
Remagnetization curve of C/Ni/N nanocomposite. The inset shows a larger scale graph.

The saturation specific magnetization of the C/Ni/N composite was measured in a magnetic field of 800 kA/m, resulting in a value of σ_s_ = 11 A·m^2^/kg. No hysteresis was observed on the magnetization curves (Figure 10). According to the analysis of the dependence of the coercive force of nickel nanoparticles on their size at room temperature, conducted in a review work [51], Ni nanoparticles become superparamagnetic when their particle size is less than 30 nm. Superparamagnets are systems consisting of magnetic particles that do not have a permanent magnetic moment due to thermal fluctuations but can exhibit magnetic properties under the influence of an external magnetic field. The absence of hysteresis in the studied composite, combined with the fact that the magnetization does not reach saturation in fields up to 300 kA/m, allows us to indirectly conclude that the size of the nickel particles in it does not exceed 30 nm.

The data obtained permit the interpretation of a broad peak with a maximum at 21 nm on the inhomogeneity distribution curve calculated from the SAXS spectra (Figure 6a). It can reasonably be concluded that this corresponds to the scattering of X-rays on nickel nanoparticles.

The nitrogen-doped C/N carbon material displays ferromagnetic properties, with a specific saturation magnetization of σ_s_ = 0.6 A·m^2^/kg and a magnetic transformation temperature of approximately 550 °C. Evidently, the graphitization of nitrogen-doped carbon results in the formation of regions of N-doped graphene [52], which, as reported by [53], can exhibit ferromagnetic properties with a high Curie temperature.

**Figure 11:**
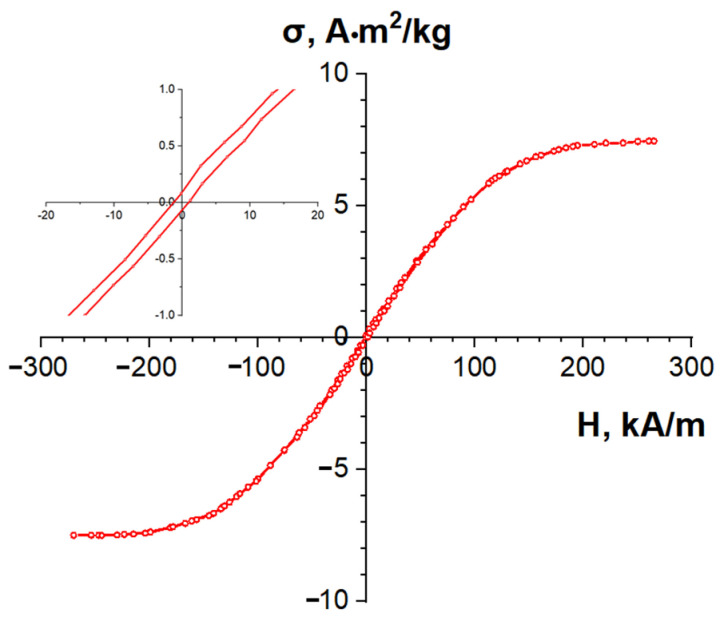
Magnetic moment hysteresis curve of the C/Ni nanocomposite. The inset shows a larger scale graph.

The C/Ni nanocomposite exhibits a saturation specific magnetization of σ_s_ = 8 A·m^2^/kg, which corresponds to a nickel content of $g_{Ni}$ = 15 wt.%, and a low coercive force of ~1 kA/m (Figure 11). The presence of a slight hysteresis may indicate that, in addition to the superparamagnetic state of nickel, a portion of the nickel particles remains in the ferromagnetic state, with a diameter greater than 30 nm.

**Figure 12:**
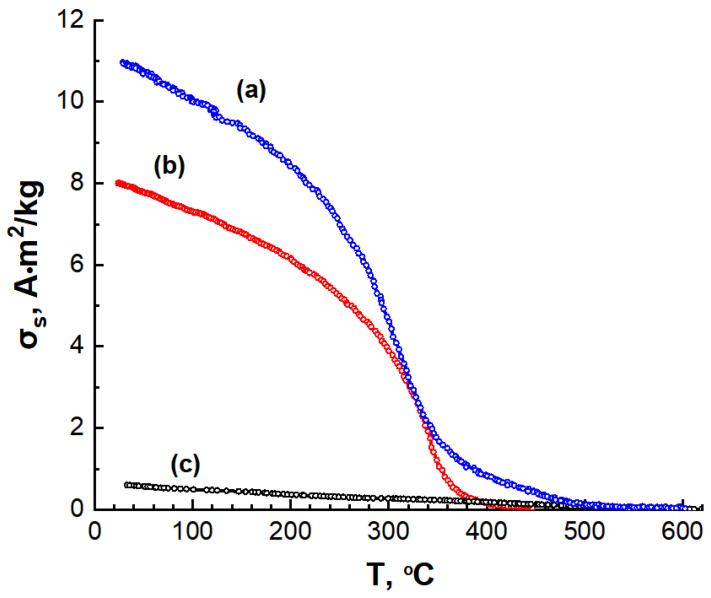
Temperature dependence of the saturation specific magnetization for C/Ni/N (**a**), C/Ni (**b**) and C/N (**c**) samples.

A phase magnetic analysis, conducted by recording the temperature dependence of the saturation magnetization in a magnetic field of 800 kA/m, revealed two magnetic phases in the C/Ni/N nanocomposite. One phase exhibits a magnetic transition temperature of approximately 350 °C, which corresponds to the nickel phase. The second phase contributes minimally to the total magnetization and exhibits a magnetic transition temperature of approximately 550 °C (Figure 12a). This second magnetic phase may be a carbon matrix resulting from nitrogen doping. The C/Ni nanocomposite and the C/N carbon material each exhibit a single magnetic phase (Figure 12b,c).

**Figure 13:**
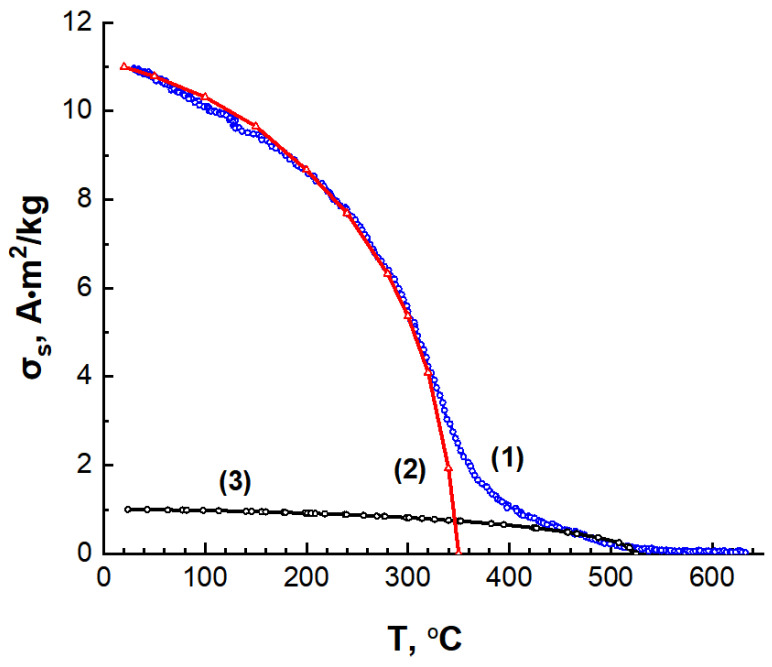
Temperature dependences of the saturation specific magnetization: 1 of C/Ni/N composite, 2, 3—model temperature dependences for Ni nanoparticles and nitrogen-containing carbon C/N, respectively.

The magnetic moment of dispersed magnetic systems obeys the additivity law, i.e., it consists of the sum of the magnetic moments of individual phases. Using the Weiss-Heisenberg model to elucidate the temperature dependence of the spontaneous saturation magnetization, we devised model temperature dependences for the individual composite phases– nickel (Figure 13, curve 2) and nitrogen-doped carbon (Figure 13, curve 3). This approach enabled us to graphically determine the contribution of each phase to the total magnetization of the composite.

According to the additivity law for the saturation specific magnetizations of the composite phases:

(3) $$\sigma_{s}=\nabla \sigma_{sNi}+\nabla \sigma_{sCN}$$

(4) $$\nabla \sigma_{sNi}=\frac{g_{Ni}}{100}\left(\sigma_{sNi}\right)$$

(5) $$\nabla \sigma_{sCN}=\frac{g_{CN}}{100}\left(\sigma_{sCN}\right)$$

where $\nabla \sigma_{sNi}$—contribution of the saturation magnetisation of nickel to the total magnetisation of the sample at a given temperature;

$\nabla \sigma_{sCN}$—contribution of the saturation magnetisation of nitrogen-containing carbon to the total magnetization of the sample at a given temperature;

$\sigma_{s}$—specific saturation magnetisation of the C/Ni/N composite;

$g_{Ni}$—mass percentage of nickel in the composite;

$\sigma_{sNi}$—specific saturation magnetization of nickel;

$g_{CN}$—mass percentage of carbon matrix in the composite;

$\sigma_{sCN}$—specific saturation magnetisation of the nitrogen-doped carbon matrix.

Supplementing Equation (3) with the mass percentage ratio:

(6) $$g_{Ni}+g_{CN}=100$$

we can calculate the specific magnetization of the doped carbon matrix as $\sigma_{sCN}$ = 1.2 A·m^2^/kg, and the composition by mass: $g_{Ni}$ = 19 wt.%, $g_{CN}$ = 81 wt.%. The calculations were based on the value of the specific saturation magnetisation of nickel for a bulk sample σ_s_ = 54.8 A·m^2^/kg [54].

## 4. Conclusions

It was found that the synthesis of nanocomposites based on hydrolysis lignin, nickel chloride and urea enables the formation of an amorphous carbon structure with nickel nanoparticles. The addition of urea contributes to the development of microporosity in the carbon material, but the effectiveness of this activator is insignificant. However, the addition of hydrolysis lignin with nitrogen derived from urea leads to the appearance of ferromagnetic behavior in the synthesized sample. When nickel chloride is added during pyrolysis, the magnetic behavior of the C/Ni/N composite is caused by superparamagnetic Ni particles smaller than 30 nm and the magnetism of the carbon matrix. The synthesized nanocomposite has no residual magnetization, so the particles will not agglomerate after exposure to an external magnetic field. During the synthesis of the C/Ni nanocomposite without nitrogen doping, Ni nanoparticles have sizes in a wide range above 30 nm. Such nanoparticles cause the occurrence of magnetic moment hysteresis.

The addition of urea during the nanocomposite synthesis further promotes better incorporation of nickel into the carbon structure. According to the results of magnetic studies, the nickel content in the C/Ni/N nanocomposite is 19 wt.% compared to 15 wt.% in the C/Ni nanocomposite.

Thanks to the porous structure of the carbon matrix with embedded nickel nanoparticles, the C/Ni/N nanocomposites can be promising catalysts, magnetically sensitive adsorbents, and active materials for electrode materials in power sources.

Thus, the conducted studies demonstrated the effectiveness of the two-stage synthesis method of carbon-nickel composites with the additional use of urea as a source of nitrogen. As a result, both the content and size of nanoparticles change. The proposed synthesis method can also be used for the synthesis of other types of carbon-metal composites. Additionally, it should be noted that the hydrolysis lignin used in the studies was directly obtained from the waste of the hydrolysis production process. Since the disposal of industrial waste is one of the important components of the transition to rational consumption and production models, the proposed synthesis method could occupy a worthy place among the methods of recycling waste into useful products.

## Figures and Tables

**Table 1:** Ratios between precursors in the synthesis of nanocomposites.

Sample	Precursors	Mass Ratio
C/Ni/N	Lignin char:NiCl_2_·6H_2_O:urea	0.75:1:0.5
C/Ni	Lignin char:NiCl_2_·6H_2_O	0.75:1
C/N	Lignin char:urea	1.5:1

**Table 2:** Average content of main chemical elements in nanocomposites based on EDX-mapping.

Sample	Weight Conc., % (Atomic Conc., %)				
	C	Ni	O	N	Si
C/Ni/N	61.93 (78.34)	20.51 (5.30)	9.31 (8.83)	5.63 (6.11)	2.62 (1.42)
C/Ni	67.01 (83.43)	19.75 (5.03)	11.20 (10.46)	-	2.04 (1.08)
C/N	71.23 (76.73)	-	17.82 (14.41)	8.25 (7.62)	2.70 (1.24)

**Table 3:** Phase composition and cell parameters in the studied samples of carbon composites.

Sample	Phase	Symmetry Group	Cell Parameters, Å
C/Ni/N	C	Amorphous	**-**
	Ni	Fm3m	a = 3.538584 ± 0.000065
	SiO_2_	P3221	a = 4.916901 ± 0.000408 c = 5.410569 ± 0.000703
C/Ni	C	Amorphous	**-**
	Ni	Fm3m	a = 3.53098 ± 0.00007
	SiO_2_	P3221	a = 4.9142 ± 0.0004 c = 5.3994 ± 0.0009
C/N	C	Amorphous	**-**
	SiO_2_	P3221	a = 4.9128 ± 0.0008 c = 5.4017 ± 0.0013

**Table 4:** Porous structure parameters of the studied samples of carbon nanocomposites.

Sample	S_BET_, m^2^/g	V, cm^3^/g	S_MP_, m^2^/g	V_MP_, cm^3^/g	S_BJH_, m^2^/g	V_BJH_, cm^3^/g
C/Ni/N	213.9	0.144	91.3	0.045	75.9	0.081
C/Ni	283.7	0.204	74.7	0.038	91.2	0.125
C/N	224.4	0.168	139.5	0.063	49.2	0.087

## Data Availability

Data are contained within the article.

## References

[1] Gupta V.K., Carrott P.J.M., Singh R., Chaudhary M., Kushwaha S. (2016). Cellulose: A review as natural, modified and activated carbon adsorbent. Bioresour. Technol..

[2] Santoso E., Ediati R., Kusumawati Y., Bahruji H., Sulistiono D.O., Prasetyoko D. (2020). Review on recent advances of carbon based adsorbent for methylene blue removal from waste water. Mater. Today Chem..

[3] Lázaro M.J., Ascaso S., Pérez-Rodríguez S., Calderón J.C., Gálvez M.E., Nieto M.J., Moliner R., Boyano A., Sebastián D., Alegre C. (2015). Carbon-based Catalysts: Synthesis and Applications. Comtes Rendus Chim..

[4] Adaikalam K., Teli A.M., Marimuthu K.P., Ramesh S., Lee H., Kim H.S., Kim H.-S. (2024). Energy Storage Application of CaO/Graphite Nanocomposite Powder Obtained from Waste Eggshells and Used Lithium-Ion Batteries as a Sustainable Development Approach. Nanomaterials.

[5] Ptashnyk V., Bordun I., Pohrebennyk V., Takosoglu J., Sadova M. (2018). Impedance investigation of activated carbon material modified by ultrasound treatment. Prz. Elektrotech..

[6] Shao H., Wu Y.-C., Lin Z., Taberna P.-L., Simon P. (2020). Nanoporous carbon for electrochemical capacitive energy storage. Chem. Soc. Rev..

[7] Cao C., Wu X., Zheng Y., Zhang L., Chen Y. (2024). Employing Manganese Dioxide and Bamboo Carbon for Capacitive Water Desalination and Disinfection. Nanomaterials.

[8] Bustillos S., Alturki A., Prentice D., La Plante E.C., Rogers M., Keller M., Ragipani R., Wang B., Sant G., Simonetti D.A. (2020). Implementation of Ion Exchange Processes for Carbon Dioxide Mineralization Using Industrial Waste Streams. Front. Energy Res..

[9] Chen W., Wan M., Liu Q., Xiong X., Yu F., Huang Y. (2019). Heteroatom-Doped Carbon Materials: Synthesis, Mechanism, and Application for Sodium-Ion Batteries. Small Methods.

[10] Ma G., Ning G., Wei Q. (2022). S-doped carbon materials: Synthesis, properties and applications. Carbon.

[11] Abdullin K.A., Gabdullin M.T., Kalkozova Z.K., Nurbolat S.T., Mirzaeian M. (2023). Symmetrical Composite Supercapacitor Based on Activated Carbon and Cobalt Nanoparticles with High Cyclic Stability and Current Load. Energies.

[12] Szymczykiewicz E., Bordun I., Maksymych V., Klapchuk M., Kohut Z., Borysiuk A., Kulyk Y., Ivashchyshyn F. (2024). Charge Storage and Magnetic Properties Nitrogen-Containing Nanoporous Bio-Carbon. Energies.

[13] Li Q., Wang Y., Wei G., Fang X., Lan N., Zhao Y., Liu Q., Lin S., He D. (2024). Spinning of Carbon Nanofiber/Ni–Cu–S Composite Nanofibers for Supercapacitor Negative Electrodes. Energies.

[14] Saini L., Patra M.K., Dhaka M.K., Jani R.K., Gupta G.K., Dixit A., Vadera S.R. (2018). Ni/graphitic carbon core–shell nanostructure-based light weight elastomeric composites for Ku-band microwave absorption applications. CrystEngComm.

[15] Deng J., Bai Z., Zhao B., Guo X., Zhao H., Xu H., Park C.B. (2021). Opportunities and challenges in microwave absorption of nickel–carbon composites. Phys. Chem. Chem. Phys..

[16] Liu X., Zhuang H. (2021). Recent progresses in photocatalytic hydrogen production: Design and construction of Ni-based cocatalysts. Int. J. Energy. Res..

[17] Chen K., Zhang S., Peng W., Qian X., Huang J. (2019). Modification of g-C_3_N_4_ quantum dots by Ni–Ni_3_C@C nanoparticles for hydrogen production. J. Phys. Chem. Solids.

[18] Meng X., Wan C., Wang Y., Ju X. (2018). Porous Ni@C derived from bimetallic Metal–Organic Frameworks and its application for improving LiBH_4_ dehydrogenation. J. Alloys Compd..

[19] An C., Deng Q. (2018). Improvement of Hydrogen Desorption Characteristics of MgH_2_ With Core-shell Ni@C Composites. Molecules.

[20] Salman M.S., Pratthana C., Lai Q., Wang T., Rambhujun N., Srivastava K., Aguey-Zinsou K.-F. (2022). Catalysis in Solid Hydrogen Storage: Recent Advances, Challenges, and Perspectives. Energy Technol..

[21] Niu L., Li Z., Sun J., Fan Z., Xu Y., Gong P., Yang S., Wang J. (2013). Hydrothermal synthesis of Ni@C core–shell composites with high capacitance. J. Alloys Compd..

[22] Vicentini R., Nunes W.G., da Costa L.H., Da Silva L.M., Freitas B., Pascon A.M., Vilas-Boas O., Zanin H. (2021). Multi-walled carbon nanotubes and activated carbon composite material as electrodes for electrochemical capacitors. J. Energy Storage.

[23] Ni Y., Jin L., Zhang L., Hong J. (2010). Honeycomb-like Ni@C composite nanostructures: Synthesis, properties and applications in the detection of glucose and the removal of heavy-metal ions. J. Mater. Chem..

[24] Gómez-Pastora J., Bringas E., Ortiz I. (2014). Recent progress and future challenges on the use of high performance magnetic nano-adsorbents in environmental applications. Chem. Eng. J..

[25] Deng Y., Xie Y., Zou K., Ji X. (2015). Review on recent advances in nitrogendoped carbons: Preparations and applications in supercapacitors. J. Mater. Chem. A.

[26] Xu X., Zhang S., Tang J., Pan L., Eguchi M., Na J., Yamauchi Y. (2020). Nitrogen-doped nanostructured carbons: A new material horizon for water desalination by capacitive deionization. EnergyChem.

[27] He G., Yan G., Song Y., Wang L. (2020). Biomass Juncus Derived Nitrogen-Doped Porous Carbon Materials for Supercapacitor and Oxygen Reduction Reaction. Front. Chem..

[28] Peng X., Luo Z., Xie H., Liang W., Luo J., Dang C., Wang A., Hu L., Yu X., Cai W. (2022). Removal of phenylarsonic acid compounds by porous nitrogen doped carbon: Experimental and DFT study. Appl. Surf. Sci..

[29] Ekman S., dos Reis G.S., Laisné E., Thivet J., Grimm A., Lima E.C., Naushad M., Dotto G.L. (2023). Synthesis, Characterization, and Adsorption Properties of Nitrogen-Doped Nanoporous Biochar: Efficient Removal of Reactive Orange 16 Dye and Colorful Effluents. Nanomaterials.

[30] Spessato L., Duarte V.A., Fonseca J.M., Arroyo P.A., Almeida V.C. (2022). Nitrogen-doped activated carbons with high performances for CO_2_ adsorption. J. CO_2_ Util..

[31] Yu F., Xiong X., Zhou L.-Y., Li J.-L., Liang J.-Y., Hu S.-Q., Lu W.-T., Li B., Zhou H.-C. (2019). Hierarchical nickel/phosphorus/nitrogen/carbon composites templated by one metal–organic framework as highly efficient supercapacitor electrode materials. J. Mater. Chem. A.

[32] Qiu Y., Yang H., Cheng Y., Bai X., Wen B., Lin Y. (2021). Constructing a nitrogen-doped carbon and nickel composite derived from a mixed ligand nickel-based a metal–organic framework toward adjustable microwave absorption. Nanoscale.

[33] Wu N., Zhai M., Chen F., Zhang X., Guo R., Hu T., Ma M. (2020). Nickel nanocrystal/nitrogen-doped carbon composites as efficient and carbon monoxide-resistant electrocatalysts for methanol oxidation reactions. Nanoscale.

[34] Bordun I., Pidluzhna A., Ivashchyshyn F., Borysiuk A., Całus D., Chwastek K. (2021). Structural and Magnetic Properties of Ni/C Composites Synthesized from Beet Pulp and Corn Stems. Magnetochemistry.

[35] Bordun I., Chwastek K., Całus D., Chabecki P., Ivashchyshyn F., Kohut Z., Borysiuk A., Kulyk Y. (2021). Comparison of Structure and Magnetic Properties of Ni/C Composites Synthesized from Wheat Straw by Different Methods. Appl. Sci..

[36] Chio C., Sain M., Qin W. (2019). Lignin utilization: A review of lignin depolymerization from various aspects. Renew. Sustain. Energy Rev..

[37] Kumar A., Kumar J., Bhaskar T. (2020). Utilization of lignin: A sustainable and eco-friendly approach. J. Energy Inst..

[38] Meek N., Penumadu D., Hosseinaei O., Harper D., Young S., Rials T. (2016). Synthesis and characterization of lignin carbon fiber and composites. Compos. Sci. Technol..

[39] Wang X., Jiang C., Hou B., Wang Y., Hao C., Wu J. (2018). Carbon composite lignin-based adsorbents for the adsorption of dyes. Chemosphere.

[40] Liu F., Wang Q., Zhai G., Xiang H., Zhou J., Jia C., Zhu L., Wu Q., Zhu M. (2022). Continuously processing waste lignin into high-value carbon nanotube fibers. Nat. Commun..

[41] Duryagina Z.A., Holyaka R.L., Borysyuk A.K. (2013). The Automated Wide-Range Magnetometer for the Magnetic Phase Analysis of Alloys: Development and Application. Usp. Fiz. Met..

[42] Sharma S.K., Vastola F.J., Walker P.L. (1997). Reduction of nickel oxide by carbon: II. Interaction between nickel oxide and natural graphite. Carbon.

[43] Gandia L.M., Montes M. (1994). Effect of thermal treatments on the properties of nickel and cobalt activated-charcoal-supported catalysts. J. Catal..

[44] Bai Z., Chen H., Li B., Li W. (2007). Methane decomposition over Ni loaded activated carbons for hydrogen production and the formation of filamentous carbon. Int. J. Hydrogen Energy.

[45] Li T., Senesi A.J., Lee B. (2016). Byeongdu Lee Small Angle X-ray Scattering for Nanoparticle Research. Chem. Rev..

[46] Honecker D., Bersweiler M., Erokhin S., Berkov D., Chesnel K., Venero D.A., Qdemat A., Disch S., Jochum J.K., Michels A. (2022). Using small-angle scattering to guide functional magnetic nanoparticle design. Nanoscale Adv..

[47] Porod G., Glatter O., Kratky O. (1982). General theory. Small-Angle X-ray Scattering.

[48] Guinier A., Fournet G. (1955). Small-Angle Scattering of X-rays.

[49] Thommes M., Kaneko K., Neimark A.V., Olivier J.P., Rodriguez-Reinoso F., Rouquerol J., Sing K.S.W. (2015). Physisorption of gases, with special reference to the evaluation of surface area and pore size distribution (IUPAC Technical Report). Pure Appl. Chem..

[50] Rouquerol J., Rouquerol F., Llewellyn P., Maurin G., Sing K. (2014). Adsorption by Powders and Porous Solids: Principles, Methodology and Applications.

[51] Krishnan K.M., Pakhomov A.B., Bao Y., Blomqvist P., Chun Y., Gonzales M., Griffin K., Ji X., Roberts B.K. (2006). Nanomagnetism and spin electronics: Materials, microstructure and novel properties. J. Mater. Sci..

[52] Zhang J., Song S., Xue J., Li P., Gao Z., Li Y., Zhang Z., Feng H., Luo H. (2018). Nitrogen-rich Porous carbon derived from biomass as high performance electrode materials for supercapacitors. Int. J. Electrochem. Sci..

[53] Miao Q., Wang L., Liu Z., Wei B., Xu F., Fei W. (2016). Magnetic properties of N-doped graphene with high Curie temperature. Sci. Rep..

[54] Coey J. (2010). Magnetism and Magnetic Materials.

